# Vaping, SARS-CoV-2, and Multisystem Inflammatory Syndrome: A Perfect Storm

**DOI:** 10.3389/fped.2021.647925

**Published:** 2021-05-12

**Authors:** Esha Brar, Anish Saxena, Claudia Dukler, Fangxi Xu, Deepak Saxena, Preneet Cheema Brar, Yuqi Guo, Xin Li

**Affiliations:** ^1^Department of Molecular Pathobiology, New York University College of Dentistry, New York, NY, United States; ^2^Department of Surgery, New York University School of Medicine, New York, NY, United States; ^3^Department of Pediatrics, New York University School of Medicine, New York, NY, United States

**Keywords:** COVID-19, multisystem inflammatory syndrome, vaping, electronic cigarette, EVALI, vaping-associated lung injury, SARS-CoV-2

## Abstract

The use of electronic cigarettes (e-cigarettes) and vaping among adolescents has risen exponentially in the last decade. E-cigarette flavors has driven adolescents to use these convenient, USB-like devices, designed to create a desired social image, while being seemingly unaware of the serious health consequences of their behavior. Vaping impacts protective pulmonary barriers by attenuating the mucociliary clearance and by increasing peribronchial inflammation and fibrosis. The recent SARS-CoV-2 (COVID-19) pandemic has been characterized by a plethora of unusual disease presentations. Among them, a unique presentation seen exclusively in children and adolescents was multisystem inflammatory syndrome (MIS-C). Seventy percent of adolescents who had MIS-C also had acute respiratory distress syndrome (ARDS), and we speculate that there may exist common denominator that links MIS-C and adolescents: the use of e-cigarettes. The virus targets the angiotensin converting receptor (ACE receptor), and studies have shown nicotine-based e-cigarettes or vaping cause oxidative stress and resulting in the upregulation of ACE2, which might worsen ARDS in MIS-C. Our mini-review highlights that adolescents using e-cigarette have alterations in their pulmonary defenses against SARS-CoV-2: an upregulation of the ACE2 receptors, the primary target of SARS-CoV-2. Their compromised immune system makes them more uniquely vulnerable to Covid-19 related MIS-C, increasing their risk for ARDS and related morbidities. Currently, studies have shown an association between MIS-C and vaping, we speculate that adolescents who vape/smoke might be especially vulnerable to serious respiratory symptoms if they develop a hyper-inflammatory state MIS-C.

## Introduction

The emergence of a novel pathogen, severe acute respiratory syndrome coronavirus-2 (SARS-CoV-2) (responsible for coronavirus disease 2019, COVID-19), has had devastating consequences, with forced quarantines, crowded hospitals with limited personal protective equipment, and thus, overwhelmed health systems across the globe. Since the first confirmed case reported in the middle of December 2019, SARS-CoV-2 has taken a significant economic and human toll. On March 12, 2020, the World Health Organization (WHO) declared COVID-19 as pandemic. An extremely virulent and pathogenic infection with an incubation period ranging from 2 to 14 days, transmitted by inhalation or contact with infected droplets, belongs to the genus Coronavirus. As per Center for Disease Control and Prevention (CDC) and WHO the symptoms vary from asymptomatic, low-grade fever, dry cough, and sore throat. Some people develop breathlessness, tiredness, body aches, fatigue, myalgia, nausea, vomiting, diarrhea, and pneumonia. Acute respiratory distress syndrome (ARDS), and multiple organ dysfunction leading to death is rare but fatality rate can be from 2 to 3% (1).

Individuals who are older than 65 years and adults with a pre-existing medical condition, such as diabetes or hypertension, are at the highest risk of serious illness upon infection; but evidence shows that this virus is relentless, sparing no specific population. In the early months of the pandemic, children and adolescents were not affected and only 2.1–7.8% cases were from children. However, severe disease outcomes have been reported amongst those approaching senescence with dampened immunity or co-morbidity of illness. Current vaccines are not available to the population under 18 years old; it is apparent that there is a growing susceptibility in adolescent smokers.

## COVID-19 Pathology

COVID-19 belongs to the family of coronaviruses, which are single-stranded RNA viruses (genera: α, β, δ, y). β coronaviruses include SARS-CoV, SARS-CoV-2 (responsible for COVID-19), and the Middle East respiratory syndrome (MERS). These viruses are named after the crown-like appearance, which is attributed to a glycosylated cell surface spike (S) protein with two functional domains: S1 and S2 (2). The mechanism of viral transmission is via aerosol and respiratory droplets, which are facilitated by close human contact. To contain the widespread infection, jurisdictions have issued a 6-foot “social distancing” rule as well as the use of face masks for the general population. Evidence shows that transmission of viruses is limited with the physical distancing of 1 m (3).

**Figure 1:**
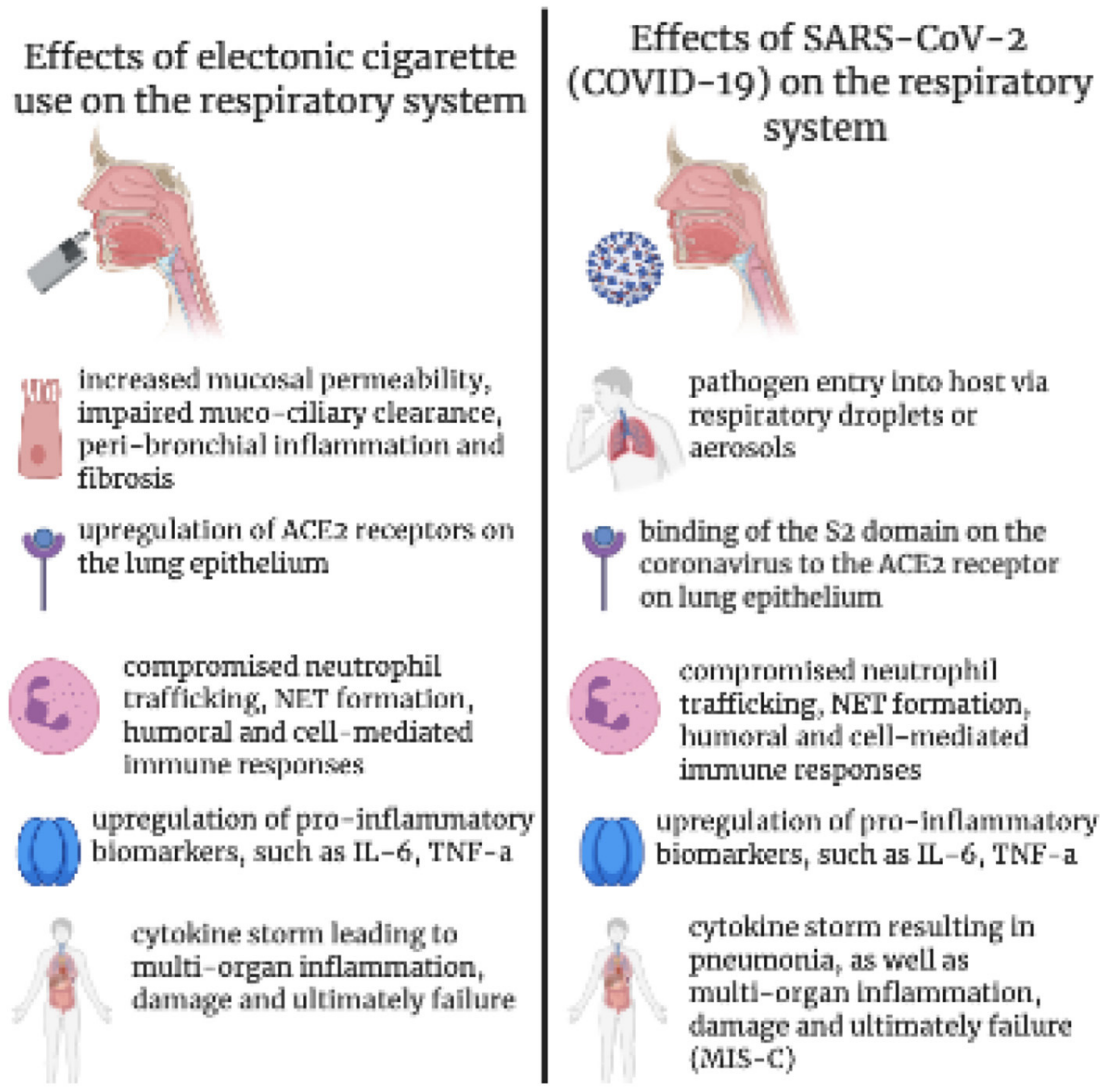
The effects of electronic cigarette use on lung and immune function in response to COVID-19 virus and similarities of pathogenicity.

Upon exposure, the virus enters its host cell and targets the angiotensin converting receptors (ACE receptors) (Figure 1), which are predominantly present in alveolar cells (4, 5). SARS-CoV-2 virus enter host-cell via ACE2. The viral spiked envelope (S2 domain) has a high affinity to the ACE2 receptor on the lung epithelium (2). Smokers (possibly including e-cigarette vapers) and individuals with hypertension/or diabetes medication have upregulation of ACE2 expression – thus rendering them susceptible to the disease (6).

While the pathogenesis of the infection caused by the virus is an area of intense investigation, evidence suggest that during SARS-CoV-2 infection there is an increase neutrophils which may contribute to severe respiratory disease with worse outcomes (7). Neutrophils affect the innate immune response with the development of neutrophil extracellular traps (NETs). NETs are a matrix of extracellular DNA, histones, and granulocyte content. These proteins and enzymes mediate prothrombotic inflammatory responses in response to viral infections. It has been demonstrated that the amount of NETs has a direct correlation with disease severity in the case of COVID-19 (7). Studies of sera from patients (n = 50) with severe COVID-19 found elevated levels of specific markers of NETs such as cell-free DNA, myeloperoxidase-DNA, and histone H3. Higher levels of NET surrogates occurred in smokers (36%) and also among those adults who required mechanical ventilation (32%). These changes in NET markers was correlated with C-reactive protein, D-dimer, and lactate dehydrogenase, and absolute neutrophil count. Overall, there is a combination of a dysregulated immune system, cytokine storm (8–10), and endothelial damage (11, 12), which determines whether patients will have mild, moderate, or severe disease outcomes.

## Kawasaki Disease and Emergence of MIS-C During the COVID-19 Pandemic

Kawasaki disease (KD), described by Tomisaku Kawasaki (case series of 50 children in Japan) in 1967, is a systemic vasculitis in children and leading causes of childhood acquired heart disease (13, 14). KD is diagnosed when there is fever >5 days and meet 4 of the 5 clinical features: (1) Erythema and cracking of the lips, strawberry tongue and erythema of the oral mucosa; (2) Bilateral conjunctivitis; (3) Erythema and edema of the hands and feet; (4) Rash; (5) Cervical lymphadenopathy (14). The pathophysiology of Kawasaki disease is as associated with autoantibodies which is the part of the immune response to a viral infection, however mechanism of autoimmunity in KD pathogenesis is still not clear particularly the functions of IgA subclass (15). The infiltration of the medium vessels by neutrophils is the characteristic necrotizing arteritis seen in KD (16). Another hallmark of the immunopathology of KD is the imbalance of IL-17 (IL-interleukin) producing T cells and regulatory T cells (17). Overall, KD can be can be hyper-inflammatory state characterized where there is elevated levels of TNF, IL-6, IL-1β, IL-17, and granulocyte colony-stimulating factor (G-CSF) (18). The COVID-19 pandemic has been characterized by a plethora of unusual disease presentations with the multisystem inflammatory syndrome in children (MIS-C) seen exclusively in children and adolescents. In April 2020, as the mayhem arising from the pandemic was becoming increasingly ubiquitous, a cluster of eight children was reported in England with a novel syndrome that was later termed MIS-C, likely related to SARS-CoV-2 infection (14). By May 2020, the CDC had published the criteria for MIS-C, which included age <21 years, fever and involvement of two or more organs, along with laboratory evidence of inflammation and infection (19). Various epidemiologic evidence suggested that SARS-CoV-2 infection may be the likely cause of MIS-C, although causality has not yet been established.

MIS-C shares similarities to KD and toxic shock syndrome. While KD presents in children 5 years old or younger, MIS-C predominantly affects adolescents and children over 7 years old and is associated with diffuse cardiovascular involvement suggestive of a generalized immune-mediated disease following the onset of COVID-19 (6). The largest targeted surveillance epidemiological study of the clinical course of pediatric MIS-C across the United States reports that 22 (12%) of 186 patients were hospitalized during March 16 and April 15, and 164 (88%) were hospitalized between April 16 and May 20. Surprisingly the peak incidence of MIS-C occurred when overall COVID-19 infection was decreasing. However, most of the patients (131 [70%]) tested positive for SARS-CoV-2 and 55 (30%) had an close contact with a person with COVID-19. The incubation period from COVID-19 symptoms to onset of MIS-C was 25 days. Rowley et al. (15) provided significant details of the ethnicity, age distribution, and gender organ involvement and suggested children have lower expression of the ACE-2 receptor and thus develop less severe pulmonary complications as compared to ARDS in adults (20, 21). Recently, the California MIS-C response group (19) analyzed the clinical presentation of 587 pediatric patients and used a statistical analysis modeling technique referred to latent class analysis to divide the patients into three groups based on similarities of presentation and to describe differing manifestations in patients who met the MIS-C case definition using serology and clinical phenotypes, such as shock, pneumonia, and organ involvement. The study included patient with average age of 8 years (range = 2 weeks to 20 years); there were 55.4% male, which included 40.5% were Hispanic or Latino. The most important comorbidity was obesity occurring in average of 30% in Hispanic, 27 in black, and 6% in white MIS-C patients. When compared this older children (range 10–15 years) (*n* = 129), had primarily respiratory system involvement (76.3%) with shortness of breath, pneumonia, and ARDS, indicating that their illnesses might have been primarily acute COVID-19 or a combination of acute COVID-19 and MIS-C. Eighty four percent of these kids were RT-PCR positivity for SARS-CoV-2 as compared to class 1 (0.5%) or class 3 (2.0%) patients (*p* < 0.01) however the case fatality rate was higher in class 2 patients (*p* < 0.01). While no reporting of the vaping/smoking habits of group #2, it is intriguing that this group of adolescents had a clinical phenotype of acute COVID-19 seen in adults (19).

Currently, little is known about the pathogenesis of MIS-C and how similar it is to KD. Recently, Consiglio et al. (22) compared the immune response of children presenting with MIS-C (*n* = 13) relative to SARS-CoV-2 (*n* = 41) infected children, children with KD (*n* = 28), and healthy children (*n* = 19) recruited before COVID-19 and found that there was hyperinflammatory response present in children with MIS-C. Lymphopenia is generally seen in COVID-19 and MIS-C and there was higher CRP and ferritin levels also in COVID-19. The levels of naive CD4+ T cell, T cell follicular helper, and increases in central memory subpopulations was higher in MIS-C as compared to KD. In adult population special in severe COVID-19 and ARDS cases there was reduction CD4+ T cells, were observed as compared to children (23). The difference between KD and severe COVID-19 and ARDS is the presentation of these terminally differentiated cells which were lower in KD as compared to COVID-19 and ARDS patients (22).

MIS-C is a systemic autoimmune disease in which T cell recognize self-antigens activating the host cell immune responses and result in inflammation. Elevated IL-6, IL-17A, and CXCL10 (C-X-C motif chemokine ligand 10) and lower IL-17A contributed the to the cytokine storm in MIS-C whereas IL-17A is significantly elevated in KD (17), suggesting a difference in the underlying immunopathology between KD and MIS-C. More mechanistic studies are underway to delineate immunopathology in MIS-C and main immune perturbation to develop new immunomodulatory based therapeutics to mitigate hyperactive inflammation and long-term tissue damage in children affected by COVID-19. Among adolescents, a silent scourge: the use of electronic cigarettes (24) should be taken into consideration in the development of both COVID-19 and MIS-C.

## The Current Prevalence of E-cigarette Usage Among Adolescents

E-cigarettes have become extremely popular among the youth on the pretense of being a safe alternative to tobacco smoke. If young Americans continue smoking at the current rate, 5.6 million of the current population under 18 years old, or one in 13, will die early of a smoking-related illness. The e-cigarette delivers nicotine by heating a vape liquid containing nicotine, flavoring agents, and various solvents into an aerosol (25). The marketing of aesthetically pleasing flavors, including candy, mango, and creme draws teens to gather recreationally and utilize the convenient, USB-like device as a means for pursuing a desired social image, yet failing to acknowledge the consequences. What smokers fail to realize is the fact that the amount of nicotine in each e-cigarette cartridge is equivalent to 200 puffs of a regular cigarette, or roughly one to three packs. A single JUUL pod can contain as much nicotine as about 20 packs of cigarettes (26). The aerosol generated contain mixtures of compound such as flavoring agents, glycerin and propylene glycol, and know toxics sentence such as heavy metals, tobacco-specific nitrosamines and harmful solvent byproducts (including formaldehyde and acrolein). All these substances can promote inflammation and harm the blood-brain barrier in a way not too dissimilar to tobacco smoke (27, 28). Vape liquids create substances cytotoxic to human pulmonary fibroblasts, lung epithelial cells, and human embryonic stem cells (29). There is evidence of oxidative stress and DNA damage, and suppression of genes related to an immune and inflammatory response in respiratory epithelial cells (Figure 1). It has been shown that e-cigarettes cause inflammation and pulmonary endothelial oxidative stress in non-smoking, healthy young subjects (30). Aside from the cellular damage initiated by the ingestion of smoke, non-inhalation behaviors leading to bacterial exposure and socioeconomic risk factors should also be taken into account.

## Contribution of E-cigarette Usage in the Development of COVID-19

ACE2 are the prime target of COVID-19 attachment and it is know that expression ACE2 is upregulated in the epithelial cells of respiratory tract of smokers and patients with smoking-associated pathologies, such as chronic obstructive pulmonary disease and idiopathic pulmonary fibrosis (31, 32). E-cigarettes or vaping which contain high amount of nicotine might contribute to overexpression of ACE2 and contribute in the serious COVID 19 associated complications (33, 34). Moreover, ACE2 is linked to nicotinic acetylcholine receptors (nAChRs), particularly alpha7nAChR receptors, further supporting the notion that vaping (nicotine) might be playing a significant role in the pathophysiology of COVID-19. Furthermore, TMPRSS2 protease important in the virus entry into the host cells are is suggested to be altered by ACE2 in vapers (2).

E-cigarette use impair mucociliary clearance, increased mucosal permeability, promote peribronchial inflammation, and fibrosis (35). It is well-established that the mucociliary epithelium and the mucous layers act as the primary line of defense against pathogens; this barrier is compromised in smokers, making them vulnerable to infections. Further, Smoking/vaping induces oxidative stress and inflammatory responses which may further contribute to COVID-19 related complication in smokers/vapers. The most common complication due to SARS-CoV2 infection – ARDS – is a result of the “cytokine storm” resulting uncontrolled release of pro-inflammatory cytokines/chemokines immune cells (6). Chronic smokers also have higher levels of expressions of IL-6, IL-17, TNF-α, and other pro-inflammatory cytokines with low expression levels of perforin and granzyme B – the two major effector proteins of natural killer (NK) and CD8 T cells (36) which further complicate COVID-19 response. This findings has been supported by studing lung autopsy of COVID-19 patients who had neutrophil infiltration in pulmonary capillaries with fibrin deposition and neutrophils into the alveolar space. There is formation of NETs which might contribute to organ and lung damage and increase the mortality in COVID-19 patients (37). NET formation, humoral and cell-mediated immune responses is also seen in smoker/vapers (2).

## COVID-19, Vaping, and Lung Inflammation

Adolescents may believe that they are “invincible” to COVID-19; this myth was quelled by a timely and groundbreaking study conducted by Gaiha et al. (38, 39). These authors performed an online national survey of 4351 adolescents and young adults aged 13–24 years in May 2020. Qualtrics panels were used to conduct social/behavioral research and multivariable logistic regression analysis assessed relationships among COVID-19-related symptoms, testing, diagnosis, cigarette and, e-cigarette use, or dual-use, sociodemographic factors, obesity, and complying with shelter-in-place.

This analysis (39) showed that the occurrence of COVID-19 was associated with the use of e-cigarettes or the use of e-cigarettes and cigarettes together. COVID-19 diagnosis was five times more likely among e-cigarettes users and seven times more likely among ever-dual-users. Further, the testing was nine times more likely among past 30-day dual-users and 2.6 times more likely among past 30-day e-cigarette only users. The rates increase in e-cigarette and cigarette users was the most important finding of this study. Risk factors for COVID-19 were males, African-Americans, and underweight adolescents. Overall, the study supports an association between COVID-19 and the use of e-cigarettes either as sole use or dual-use with cigarettes among the youth (39).

The tendency for smokers to be infected by the COVID-19 virus is unlikely to be a coincidence. It is known that a compromised immune system and damaged lung epithelia make this population susceptible to infection. They often exhibit exacerbated symptomatology and longer duration upon infection. Similarly, vaping is also detrimental to lung health and the immune system. E-cigarette or vaping product use associated lung injury (EVALI)' is a condition seen in vapers include acute lung injury, acute fibrinous pneumonitis, diffuse alveolar damage, or pneumonia accompanied by bronchiolitis (2). In contrast, e-liquid aerosol contents can also interact with the alveolar surface active material, to induce innate immune responses in electronic cigarette users, hence making smokers more prone to developing COVID-19 symptomatology. One of these can potentially include Vitamin E Acetate, part of the aerosol of a vaping device that can lead to permanent scarring of the lungs, and along with other metals and lead, it can start to accumulate and build up within the lungs, leading to persistent cough or cold. Smoking/vaping modulate the immune response facilitating virus entry and survival which can result in hyper active immune response triggered by “cytokine storm” in the host. This sequence of events ultimately results in damaged lung tissue and exacerbated reaction to another pathogen (2).

Martin et al. (40) studied nasal scraping among smokers, demonstrating similar changes in the expression of immune-related genes. Genes that had decreased expression in cigarette smokers (*n* = 53) were also decreased in e-cigarette smokers. Moreover, vaping e-cigarettes was also associated with suppression of other large number of unique genes (*n* = 305) through downregulation of key transcription factors. Rebuli et al. (41) have recently shown that the SMPD3, NOS2A, and KLRB1 genes are downregulated in cigarette smokers, and the MR1, NT5E, and HRAS genes are downregulated in e-cigarette users. Reidel et al. (42) showed that changes in innate defense proteins are associated with significantly elevated elastase and matrix metalloproteinase-9 in e-cigarette users. E-cigarette users' sputum had increased NET-related proteins, such as myeloperoxidase, azurocidin, and protein-arginine deiminase 4, suggesting an association of vaping and organ damage in COVID-19. With vaping being a great attraction for adolescents, the effects of vaping can result in an increased amount of vulnerability in this age group.

## Discussion

The use of e-cigarettes has a multitude of adverse effects on both local and peripheral levels. They increase epithelial permeability caused by tight junction damage, stripping the mucociliary epithelial barrier and leading to a compromised defense mechanism against pathogens, peribronchial inflammation, oxidative stress, and ultimately, a more detrimental immune response (Figure 1). While inflammation is necessary for the presence of a foreign body or disease, it is a meticulous process. In normal individuals, the viral infection is controlled or checked by immune cells, cytokine response, goblet and nasal epithelial/ciliated and oral mucosal cells, however smoking or vaping can weaken these defenses against viral replication. HuangFu et al. (43) showed that smoking affects specific serine phosphorylation-dependent ubiquitination and degradation of the Interferon Alpha and Beta Receptor subunit of the Type I IFN receptor, and this affects TNF signaling, making the lung epithelium more vulnerable to viral entry. Modestou et al. (44) have reported that cigarettes affect the markedly inhibited IFN-gamma-induced Stat1 phosphorylation, indicating that smoking altered type II interferon signal transduction. These studies show that alteration in interferon signaling affects the entry of common viruses like respiratory syncytial and rhinovirus.

Studies have highlighted that SARS-CoV2 has a deleterious effect on smokers. The inflammatory state of MIS-C reported in adolescents related to COVID 19 demonstrated similar laboratory features of hyperinflammation to that seen in smokers who COVID-related ARDS. It is the timing of onset in relation to SARS-CoV-2 infection, and the similarities to adults with COVID-19 in terms of the disease pattern which supports the hypothesis that MIS-C is a consequence of immune-mediated injury triggered by SARS-CoV-2 infection. Thus, we speculate that the use of e-cigarettes increases the likelihood of MIS-C-related respiratory complications.

Aside from the biological cascade in response to smoking e-cigarettes, the physical action of smoking behavior, as well as the environmental and societal circumstances, might also be a contributing factor to users' susceptibility to COVID-19 when compared to a non-smoker. There are several explanations as to why both dual-use and e-cigarette use was associated with COVID-19 infection. Heightened exposure to nicotine and other chemicals in e-cigarettes adversely affects lung function, is further complicated by high viral exposure due to physical behavior (such as frequently touching their mouth with hands while using their vaping devices), sharing smoking devices, and ultimately, the presence of a damaged immune system. These factors make specific populations more susceptible to viral attacks, exacerbated symptoms, and difficulty in antibody development. Some racial/ethnic groups, especially among African American, Hispanic, and multiracial youth, are at higher risk for COVID-19, with smoking and vaping other contributing factors include living condition where social distancing is a challenge, economic stress, work environments where working from home is less feasible is further complicated by less access to health care (38). These findings show that vaping is causal in increasing the risk for COVID-19 by dampening immune function, in turn promoting the development of complications, such as MIS-C.

The strengths of this mini-review are that, for the first time, it highlights the possibility that among MIS-C adolescents, vaping might be a risk factor for respiratory symptoms, even though the literature on MIS-C does not report smoking history. Gaiha et al. (38, 39) showed that smokers, especially dual users over the last 30 days, were nine times more likely to be tested for COVID-19, and that their behaviors associated with smoking increased infection risk. Our review raises the importance of investigating this question further in adolescent smokers who might have a greater risk for COVID-19 infection. Moreover, major vaccine-producing companies are going to start clinical trials of the COVID19 vaccine in 12 years and older children in the near future, and this review will provide much timely information on vaping and COVID in this young population.

A limitation of this study is the lack of literature support to establish a causative connection between COVID-19 and MIS-C, as there is only an indirect association based on surveys by Gaiha et al. (38) showing that adolescents who smoke are more likely to experience COVID19-related symptoms and possibly be at increased risk for MIS-C.

At the time of writing this review there was great success of COVID-19 vaccine development, both protein-based and gene-based, the COVID-19 pandemic is expected to be close to ending. Interestingly, the phase I clinical trial Safety and Immunogenicity Study of 2019-nCoV Vaccine (mRNA-1273) for Prophylaxis of SARS-CoV-2 Infection (COVID-19) conducted by the National Institute of Allergy and Infectious Diseases (NIAID) (NCT04283461) has excluded any subjects ≥ 56 years of age with a history of chronic smoking within the prior year or current smoking or vaping. It is possible that the damage caused by smoking or vaping on one's immune system might affect the response to or even the efficacy of the vaccine. More scientific investigations are required to assess the impact of vaping on COVID-19 infection and vaccination among the young. However, refraining from vaping or smoking is undoubtedly a wise choice. COVID-19 has been able to strike the world relentlessly, affecting various groups. We hope to find that the group of adolescents who actively vape will begin to reconsider their habits due to the presence of a highly infectious virus; and that the number of e-cigarette and cigarette-related illnesses will decrease. From a health policy perspective, regulating the use of e-cigarettes during this pandemic and the distribution of youth-focused COVID-19 prevention messaging to e-cigarette and dual users is important. Prospective longitudinal studies focused on COVID-19 related serious complications like MIS-C in children and adolescents with a smoking history be elucidated and compared to that reported in adult smokers.

## Author Contributions

EB, AS, and CD: formal analysis, writing—original draft, and writing—review and editing. FX and YG: writing—review and editing. PC: developed the idea and edited the first draft. DS and XL: funding acquisition and developed the idea. XL: administration, supervision, and validation. All authors contributed to the article and approved the submitted version.

## Conflict of Interest

The authors declare that the research was conducted in the absence of any commercial or financial relationships that could be construed as a potential conflict of interest.
